# Development of the use of primary health care emergency departments after interventions aimed at decreasing overcrowding: a longitudinal follow-up study

**DOI:** 10.1186/s12873-022-00667-9

**Published:** 2022-06-14

**Authors:** Marja Liedes-Kauppila, Anna M. Heikkinen, Ossi Rahkonen, Mika Lehto, Katri Mustonen, Marko Raina, Timo Kauppila

**Affiliations:** 1 Department of Public Health, University of Helsinki, Finland, Biomedicum 2, Tukholmankatu 8 B, SF-00014 Helsingin yliopisto, Helsinki, Finland; 2 Department of Oral and Maxillofacial Diseases, Head and Neck Center, University of Helsinki, Helsinki, Finland; 3 Department of General Practice and Primary Health Care, University of Helsinki and Helsinki University Hospital, Helsinki, Finland; 4 Vantaa Health Centre, Vantaa, Finland

**Keywords:** Emergency department, Primary care, Practice management

## Abstract

**Background:**

This study, conducted in a Finnish city, examined whether decreasing emergency department (ED) services in an overcrowded primary care ED and corresponding direction to office-hours primary care would modify service usage for specific gender, age or diagnosis groups.

**Methods:**

This was an observational retrospective study carried out by gradually decreasing ED services in primary care. The interventions aimed at decreasing use of EDs were a) application of ABCDE-triage combined with public guidance on the proper use of EDs, b) closure of a minor supplementary ED, and finally, c) application of “reverse triage” with enhanced direction of the public to office-hours services and away from the remaining ED The annual number of visits to office-hours primary care GPs in different gender, age and diagnosis groups (International Classification of Diseases (ICD − 10) were recorded during a 13-year follow-up period.

**Results:**

The total number of monthly visits to EDs decreased slowly over the whole study period. This decrease was similar in women and men. The decrease was stronger in the youngest age groups (0–19 years). GPs treated decreasing proportions of ICD-10 groups. Recorded infectious diseases (Groups A and J, and especially diagnoses related to infections of respiratory airways) tended to decrease. However, visits due to injuries and symptomatic diagnoses increased.

**Conclusion:**

Decreasing services in a primary health care ED with the described interventions seemed to reduce the use of services by young people. The three interventions mentioned above had the effect of making the primary care ED under study appear to function more like a standard ED driven by specialized health care.

## Introduction

Overcrowding of Emergency Departments (EDs) is common in several countries and therefore health authorities have tried to remedy the problem in several ways [1]. In the city of Vantaa, due to constant overcrowding of primary care EDs the health authorities initiated three different actions to guide non-urgent patients away from the local primary care ED during the years 2004–2008. The actions were: application of ABCDE-triage combined with public guidance on the proper use of EDs [2], closure of a minor supplementary ED [3], and, finally, application of “reverse triage” with enhanced guiding of the public to office-hours services and away from the remaining ED [4]. The strategy was that those patients who did not require doctor services in EDs would be guided to office-hours GPs in the local primary care by the primary care system itself [5]. This strategy did not work as planned: patients were not directed to office-hours physicians [5].

There are many studies relating to the ways in which the use of primary care physicians may be utilised to decrease overcrowding in EDs [6]. Usually, the researchers have looked for a positive impact on ED utilization. Similarly, the majority of studies have reported length of visit, departure without being seen by medical staff, patient satisfaction, time to provide initial assessment, ED workup time, departure contrary to medical advice, patient safety and departure before completion of service. Following changes in recoded diagnoses is one additional way to answer the question: Do we really change the types of patients when we perform all kinds of interventions to reduce ED overcrowding? Furthermore, as patients were not directed to office-hours physicians after the present interventions [5] it became important to study which patient groups in the ED were most affected by these reductions. In this longitudinal follow-up study, we examined how this decrease in ED services impacted on the access of different gender, age and diagnosis groups to the ED.

## Methods

### Setting and design

The present study is a retrospective longitudinal follow-up study. It was performed in office-hours services in the primary health care of the fourth largest city of Finland. In Vantaa there were about 210,000 inhabitants in 2014. Visits to primary care EDs were studied. As everywhere in Finland, primary care is non-profit and municipalities, which fund this activity with taxes, maintain it. The ED system had two departments. The first evaluation was usually performed by the primary care ED system and if treatment in secondary care was necessary the patients were referred to the ED of the university clinic of Helsinki University (HUS) in the Peijas or Meilahti hospitals. Thus, the low acuity patients came first to the primary care ED system of the city of Vantaa [2–4].

The register keepers (the social and health authorities of Vantaa) and the scientific ethical board of Vantaa City (TUTKE) granted permission (VD/8059/13.00.00/2016) to carry out the study. This study was implemented using the patient information system and anonymized patient data, thus without identifying the patients or physicians. According to Finnish law regarding register studies (https://rekisteritutkimus.wordpress.com/luvat-ja-tietosuoja/), the study participants did not need to sign a Statement of Informed Consent because the study was retrospective, anonymized, based on patient information charts, and the investigators did not contact the participants.

### ED interventions

Three different interventions were initiated in the primary care ED system of Vantaa. Strategically, they were planned simultaneously but carried out gradually by the administration of the primary care of the city of Vantaa. First, an ABCDE-triage system combined with public guidance on the proper use of EDs was introduced in the main primary care ED of the city of Vantaa on 1.1.2004 [2]. Leaders responsible for the implementation of the intervention were chosen. The project workers analysed the process and patients in need of special attention were identified based on interviews with health policy specialists. These were elderly people, children and people suffering from mental illness or drug abuse. A discussion was raised in the media around these services and information was delivered both to professionals and the public and, thereby, the impact of introducing the ABCDE-triage tool in emergency services was also enhanced by increasing public knowledge about the issue. Guidelines were introduced for the staff when performing the triage; new practices were established by training, and through motivation and encouragement. The general public was informed of the project through the media and the information focused on the transparency of the system. Internet, local print media, radio and bulletins were used. The aim of the project group was to publish as much information as possible related to the changes to keep the population, all organizations associated with the project and the staff fully informed. The objective of this massive information campaign was to guide non-acute patients to appropriate daytime services. Feedback was actively gathered, both from patients and the staff, with questionnaires and interviews. Numbers of visits to doctors and nurses, number of patients treated, triage groups, waiting times and diagnoses were frequently assessed. The staff was encouraged to follow the guidelines and provide leaders with useful information. Follow-up meetings were organized in order to discuss the implementation process and problematic patient cases. ABCDE-triage was performed by an experienced nurse. This took place in the first line of the emergency service before the patient attended the doctor. The patients were triaged subjectively by the nurse, with classification described in detail in [2]. The group E-patients were able to stay and wait if they wanted to see a doctor, even though the triage nurse had explained to the patient that his/her case was assessed to group E (non-acute). If the status of the patient altered in the waiting room a re-triage was performed. Consequently, those patients who judged by themselves that their condition did not require emergency actions did not arrive at the ED when they realised that they would be forced to wait a long time to see a doctor. Secondly, a small suburban supplementary ED (Myyrmäki ED) was closed in the western part of Vantaa [3]. This intervention was performed in June 2005. Vantaa is divided into five health care districts. The main primary care ED, Peijas, is located in Korso-Koivukylä district (population about 46,000 inhabitants in 2005: 19 km from Myyrmäki ED). In the eastern part of Vantaa city there are two other districts, Tikkurila (the economic and administrative centre of Vantaa city, about 47,000 inhabitants; 5 km from Peijas ED, 15 km from Myyrmäki ED) and Hakunila-Länsimäki (about 28,000 inhabitants; 8 km from Peijas ED, 20 km from Myyrmäki ED). The two remaining health care areas are both located in the western part of Vantaa. The smaller primary care ED was located in Myyrmäki district (34,000 inhabitants; 17 km from Peijas ED), and there is also the neighbouring Martinlaakso district (26,000 inhabitants; 19 km from Peijas ED, 3 km from Myyrmäki ED). Since both primary and secondary care are provided in the ED at Peijas Hospital it is defined as a ‘combined ED’. It is equipped with out-of-hours laboratory and X-ray facilities, and primary care ED services are carried out there only out of office hours. As a comparison, the primary care ED in Myyrmäki resembled a traditional Finnish primary health care out-of-hours unit, did not provide specialist care, and the laboratory and X-ray facilities were available only during office hours. This closed ED was not open during the night-time but only in the evenings and at weekends. Performing this intervention meant that patients who originally sought help from a small nearby ED had to travel an average of 17 km more to reach the main primary care ED, compared to before the closure. Consequently, those patients who judged by themselves that it was not worth the extra burden of travelling to the remaining ED to get their health problem treated immediately in emergency did not appear in the ED at all. Thirdly and finally, a tight “reverse triage”, based on ABCDE-categorization was introduced in the remaining primary care ED [4]. This meant that those patients who were triaged to group E did not meet a doctor in the ED as they had been able to do after the original ABCDE-triage [2]. Instead, they were given self-treatment advice or instructed in how to book a time with their own office-hours primary care physicians. Consequently, those patients who judged by themselves that their health issue did not require emergency actions did not arrive at the ED. In practice, these interventions led to a situation where the amount of doctor visits in the primary care ED system decreased by almost 50% [2–5].

### Primary and secondary measures

The data were obtained from Graphic Finstar patient chart system (GFS, Logica LTD, Helsinki, Finland). The follow-up period was 1.1.2002–31.12.2014. The report generator of the GFS-system provided yearly figures for the number of ED visits in different gender and age groups (0–19, 20–64 and 65+ years). This was the main measure for analysis in the present study. The other measure from the patient chart was the rate of change (in number or proportion/year) of different ICD-10 (International Classification of Diseases 10th edition) diagnoses [7] recorded. The ICD-10 diagnoses were collected and examined at an accuracy of initial number and three first digits. The twenty most common diagnoses in the office-hours services were studied in detail.

### Statistical analyses

The rates and the directions of change in numbers of all studied parameters, i.e. the rates of development in different patient groups, were analyzed using regression analysis followed by t-test (GLM procedure of SigmaPlot 10.0 Statistical Software, Systat Software Inc., Richmond, CA, USA) [8–10]. Thus, the GLM allowed us to count the mean slope (cofactor a) of the development of the amount of physician visits (visits/year) and its standard error of the mean (SEM) during the follow-up. Analogously, the rate of change in number and proportions (change in number or %/year) of different ICD-10 diagnoses were counted. The comparisons with t-test were then performed to determine whether there were statistically significant changes in in the slopes. Similar comparisons with one-way Analysis of Variance (ANOVA followed by Bonferroni method as a post hoc test) were also performed between the slopes of different study groups to detect whether these groups differed from each other. *P* value < 0.05 was considered as statistically significant.

## Results

### Effect of age

**Fig. 1:**
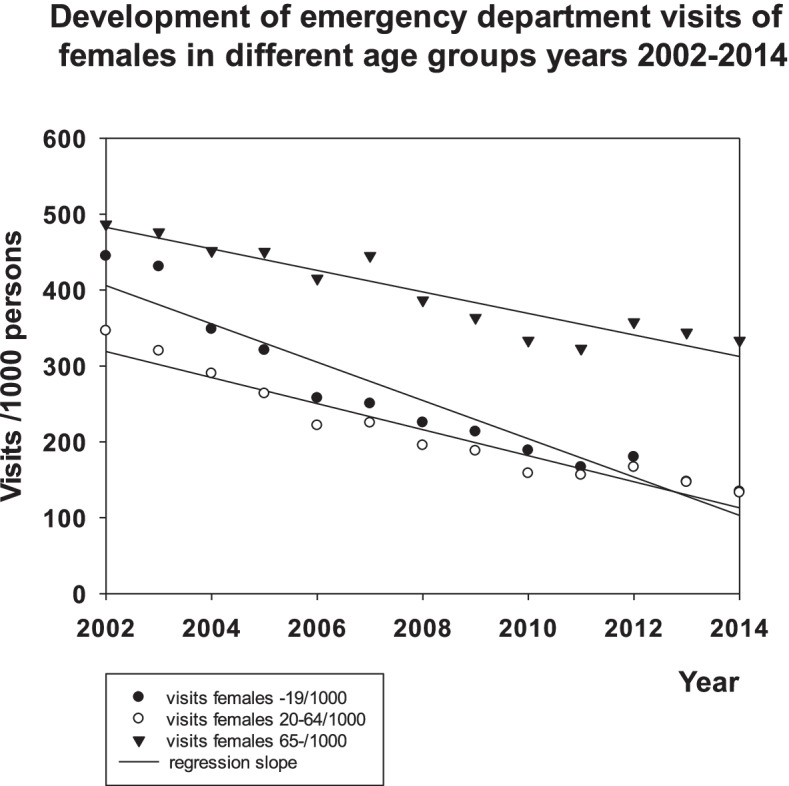
Development of visits of females to EDs in Vantaa 2002–2014

**Fig. 2:**
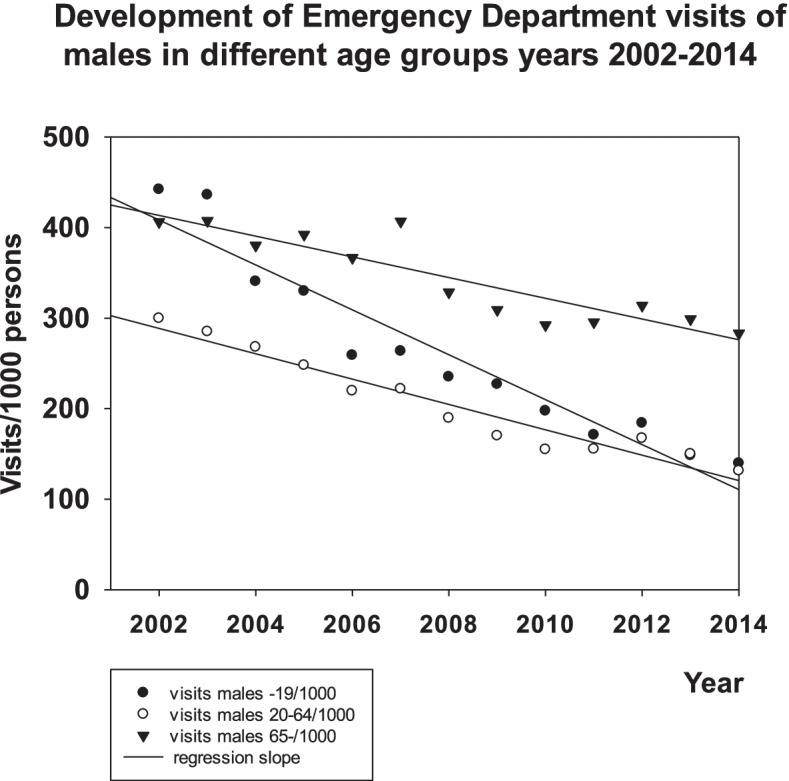
Development of visits of males to EDs in Vantaa 2002–2014

The decrease in the rate of visits to the ED was − 25.3 ± 2.3 visits/1000 persons/year (mean ± SEM.: *p* < 0.001, t-test) in females aged 0–19 and did not differ statistically from the respective rate of males in the same age group (− 24,8 ± 2.2: p < 0.001). The decrease was higher in the two youngest groups and it differed (p < 0.001, ANOVA) from the respective rates of decrease (all p < 0.001, t-test) of females aged 20–64 (− 17.2 ± 1.5), 65+ (− 14.2 ± 1.6) and males aged 20–64 (− 14.0 ± 1.1) or 65+ (− 11.4 ± 1.6). There were no differences in these rates between the female and male age groups 20–64 or 65+ (see Figs. 1 and 2).

### Change in diagnostics

During the follow-up of the interventions the recorded proportions of ED visits related to infections (main ICD-10 groups A and B), respiratory diseases (group J) and musculoskeletal diseases (group J) decreased (Table 1). In line with this general observation regarding the decrease in infectious diseases, both the recorded proportions and absolute numbers of visits related to Acute upper respiratory infections of multiple and unspecified sites, Suppurative and unspecified otitis media, Acute bronchitis, Acute tonsillitis, Conjunctivitis, and Acute sinusitis decreased (Tables 2 and 3).

**Table 1:** Development of the proportions of main ICD-10 diagnosis groups in 2002–2014 in primary care EDs. The arrows show the direction of the observed change

ICD-10	Contents of diagnosis group	Rate of change (mean ± SEM, %/year)	***p-***value
**A**	Intestinal infectious diseases, bacterial infections and viral infections of central nervous system	−0,0540 ± 0,0235	**0,042↓**
**B**	Other infections	−0,0337 ± 0,00515	**< 0,001↓**
**C**	Malignant neoplasms	0,0136 ± 0,00419	**0,008↑**
**D**	Other neoplasms and Carcinoma in situ	0,00538 ± 0,00309	n.s.
**E**	Endocrine nutritional and metabolic diseases	0,0514 ± 0,00555	**< 0,001↑**
**F**	Mental and behavioural disorders	0,443 ± 0,0434	**< 0,001↑**
**G**	Diseases of the nervous systems	0,0449 ± 0,0174	**0,026↑**
**H**	Diseases of the eye and the adnexa, and the ear and mastoid process	−0,721 ± 0,0631	**< 0,001↓**
**I**	Diseases of the circulatory system	0,210 ± 0,0427	**< 0,001↑**
**J**	Diseases of the respiratory system	-1708 ± 0,159	**< 0,001↓**
**K**	Diseases of the digestive system	0,0597 ± 0,0250	**0,036↑**
**L**	Diseases of the skin and subcutaneous tissue	0,00245 ± 0,0248	n.s.
**M**	Diseases of the musculoskeletal system and connective tissue	−0,203 ± 0,0443	**< 0,001↓**
**N**	Diseases of genitourinary system	0,166 ± 0,0226	**< 0,001↑**
**O**	Pregnancy, childbirth and puerperium	−0,00530 ± 0,00227	**0,039↓**
**P**	Certain conditions originating in the perinatal period	−0,000391 ± 0,000469	n.s.
**Q**	Congenital malformations, deformations and chromosomal abnormalities	−0,000793 ± 0,000637	n.s.
**R**	Symptoms, signs and abnormal clinical and laboratory findings, not elsewhere classified	1050 ± 0,0958	**< 0,001↑**
**S**	Injury, poisoning and certain other consequences of external causes, single body region	0,451 ± 0,0914	**< 0,001↑**
**T**	Injuries to multiple or unspecified body regions as well as poisoning and certain other consequences of external causes.	0,0647 ± 0,0345	n.s.
**V**	Transport accidents	0,0122 ± 0,00669	n.s.
**W**	Other external causes of accidental injury	0,0529 ± 0,00916	**< 0,001↑**
**X**	Exposure to burning substances and related threads, venomous animals and plants, noxious substances and forces of nature. Intentional self-harm and assault	0,0349 ± 0,00621	**< 0,001↑**
**Y**	Events of undetermined intent, legal interventions and operations of war, complications of medical care, sequelae of external causes of morbidity and mortality	0,0122 ± 0,00464	**0,024↑**
**Z**	Factors influencing health status and contact with health services	0,0513 ± 0,0189	**0,020↑**

**Table 2:** Development of the proportions of the twenty most common ICD-10 diagnoses in 2002–2014 in primary care EDs. The arrows show the direction of the observed change

ICD-10 code	Name of diagnosis	Rate of change (mean ± SEM, %/year)	***p-***value
**J06**	Acute upper respiratory infections of multiple and unspecified sites	−0,00618 ± 0,000740	**< 0,001↓**
**R10**	Abdominal and pelvic pain	0,00312 ± 0,000284	**< 0,001↑**
**H66**	Suppurative and unspecified otitis media	−0,00430 ± 0,000305	**< 0,001↓**
**M54**	Dorsalgia	−0,00195 ± 0,000175	**< 0,001↓**
**S01**	Open wound of head	0,000880 ± 0,000246	**0,004↑**
**J20**	Acute bronchitis	0,00365 ± 0,000362	**< 0,001↑**
**A09**	Other gastroenteritis and colitis of infectious and unspecified origin	−0,00101 ± 0,000133	**< 0,001↓**
**F10**	Mental and behavioural disorder due to use of alcohol	0,00261 ± 0,000257	**< 0,001↑**
**R07**	Pain in throat and chest	0,00148 ± 0,000106	**< 0,001↑**
**S93**	Dislocation, sprain and strain of joints and ligaments at ankle and foot level	−0,000614 ± 0,000156	**0,002↓**
**S61**	Open wound of wrist and hand	−0,0000183 ± 0,000186	n.s.
**N30**	Cystitis	0,000297 ± 0,000108	**0,019↑**
**J03**	Acute tonsillitis	-0,00270 ± 0,000243	**< 0,001↓**
**H10**	Conjunctivitis	-0,00156 ± 0,000294	**< 0,001↓**
**J01**	Acute sinusitis	-0,00284 ± 0,000324	**< 0,001↓**
**M79**	Other soft tissue disorders, not elsewhere classified	0,00159 ± 0,000177	**< 0,001↑**
**S06**	Intracranial injury	0,0000570 ± 0,000116	n.s.
**R53**	Malaise and fatigue	0,00169 ± 0,000160	**< 0,001↑**
**R06**	Abnormalities of breathing	0,000741 ± 0,000164	**< 0,001↑**
**S52**	Fracture of forearm	0,00108 ± 0,000236	**< 0,001↑**

**Table 3:** Development of the absolute values of the twenty most common ICD-10 diagnoses in 2002–2014 in primary care EDs. The arrows show the direction of the observed change

ICD-10 code	Name of diagnosis	Rate of change (mean ± SEM, N/year)	***p-***value
**J06**	Acute upper respiratory infections of multiple and unspecified sites	125,637 ± 33,307	**0,003↓**
**R10**	Abdominal and pelvic pain	127,802 ± 20,857	**< 0,001↑**
**H66**	Suppurative and unspecified otitis media	0,00864 ± 0,00162	**< 0,001↓**
**M54**	Dorsalgia	22,176 ± 15,721	n.s.
**S01**	Open wound of head	46,604 ± 10,158	**< 0,001↑**
**J20**	Acute bronchitis	80,484 ± 16,028	**< 0,001↓**
**A09**	Other gastroenteritis and colitis of infectious and unspecified origin	11,791 ± 8672	n.s.
**F10**	Mental and behavioural disorder due to use of alcohol	90,011 ± 9114	**< 0,001↑**
**R07**	Pain in throat and chest	0,0147 ± 0,00193	**< 0,001↑**
**S93**	Dislocation, sprain and strain of joints and ligaments at ankle and foot level	2396 ± 5381	n.s.
**S61**	Open wound of wrist and hand	13,813 ± 5642	**0,032↑**
**N30**	Cystitis	22,154 ± 6185	**0,004↑**
**J03**	Acute tonsillitis	60,918 ± 6996	**< 0,001↓**
**H10**	Conjunctivitis	31,341 ± 6078	**< 0,001↓**
**J01**	Acute sinusitis	67,214 ± 11,802	**< 0,001↓**
**M79**	Other soft tissue disorders, not elsewhere classified	55,604 ± 8155	**< 0,001↑**
**S06**	Intracranial injury	12,148 ± 5756	n.s.
**R53**	Malaise and fatigue	0,0152 ± 0,00179	**< 0,001↑**
**R06**	Abnormalities of breathing	29,582 ± 6004	**< 0,001↑**
**S52**	Fracture of forearm	39,846 ± 8320	**< 0,001↑**

Simultaneously, there was an increase in the recorded proportions of visits related to endocrine diseases (ICD-10 group E), mental disorders (group F), circulatory system diseases (group I), genitourinary diseases (group N) and various types of injuries (groups S, T, W, X and Y). The increase in different injuries was especially remarkable in the recorded proportions and absolute numbers of visits related to Open wounds of head and Fractures of forearm (Tables 2 and 3).

The proportion of recorded symptomatic diagnoses (group R) increased strongly. This increase was seen especially in the recorded proportions and absolute numbers of visits related to Abdominal and pelvic pain, Pain in throat and chest, Malaise and fatigue and Abnormalities of breathing (Tables 2 and 3).

## Discussion

The number of visits to EDs decreased during the follow-up of the interventions. This decrease was most prominent in the youngest age groups. Especially, the proportions of recorded infectious diseases (Groups A, B and J) decreased. Particularly, diagnoses related to mild infections of respiratory airways decreased. Interestingly, the effects of interventions on the prevalence of different injuries varied, although generally the proportions and absolute numbers of injuries increased. The proportions and numbers of symptomatic diagnoses increased.

The implementation of the ABCDE-triage system for assessing patient acuity at Peijas combined ED reduced the number of patient visits to GPs of the ED by 8 % [2], closing the minor suburban ED brought the reduction in visits to 15–20% [3] and further applying “reverse triage” brought the total reduction in visits to about 25% [4]. No increased mortality was found [5]. Providing enough information about the ED changes to the population proved to be important for the success of the implementation of the present interventions [2–4].

The decreased rate in the use of primary care EDs in the youngest people (0–19 years) is understandable. There are earlier reports suggesting that primary health care ED services are often used by the younger age groups [11, 12]. Especially low acuity visits to EDs seem to be a feature of very young age groups (< 10 y) and late teenagers (18–19 years) [13]. Interestingly, social deprivation does not seem to influence this pattern of ED use [13]. The reasons for this are many. According to a survey study with ED patients, young age groups may differ in their expectations regarding the purpose of out-of-hours services and accessibility and they may have other objectives than plain clinical urgency when they seek help [14]. Furthermore, in a multicenter survey of patients from an urban health region, distance to a specific ED was the most important reason for choosing that ED suggesting that convenience factors play a significant role when deciding to use ED services [15]. There is support for this view from other studies analyzing primary care out-of-hours calls and visits concerning child patients [16] and young adults [17] as well as from qualitative studies regarding treatment of small children in out-of-hours services [18]. Nevertheless, the use of ED services in the youngest age group is strongly regulated by their adult parents. The adults did not seem to reduce the number of their own visits as strongly. Based on this and former data [14–18] the adults may change their estimation of the acuity of the health problems of children and youngsters more readily than of their own, if the inconvenience of visiting the ED is increased, with longer waiting times and prolonged distances to reach the ED. Altogether, previous findings have shown that if restrictions of access to primary health care EDs occur, the youngsters, who use these services a lot, reduce their ED visits more than other people.

Where did the patients go if they did not come to the primary care ED? Some patients were redirected to a single speciality ED after applying ABCDE-triage [2] and there may have been some marginal spillover to private sector primary care after closing the minor suburban primary care ED [3]. Some patients were redirected to both a single speciality ED and private sector primary care after starting the “reverse triage” [4]. However, the number of patients who were redirected to other practices was less than the reduction in the number of visits to the primary care ED [2–4]. Neither was there a spillover to public office-hours primary care [5, 18]. As there was no change in the secondary ED functions during the follow-up period the people of Vantaa might have changed the way they estimated the acuity of their health problems.

Access to individual patient data would have given detailed information about the safety of the combination of ED-interventions that were applied. We had only mortality data, which gave a crude but definite estimation of the safety of the studied interventions [3, 4]. The overall mortality in Vantaa increased during the present follow-up period because of ageing of the population [19]. However, in the studied age groups 20–64 and 65+ the mortality tended to decrease [18]. Only a slight plateauing of the decreasing monthly mortality was observed in the oldest age group (65+) after 2008, the year of the implementation of “reverse triage”, [5] but analogous plateauing was observed in the mortality of the whole Finnish 65+ age group in the period 2008–2014 [20]. Thus, no lethal safety hazards were found in this analysis.

The finding that the proportions and numbers of simple infections in the ED decreased is in line with the aims of the interventions applied [2–4]. We knew that at least about 30% of the diagnoses done in the present kind of primary care ED system and office-hours primary care were the same and that office-hours primary care might therefore have provided better continuation of treatment for these patients than the ED [21]. Furthermore, when diagnoses in EDs and primary care doctor driven emergency systems have been compared, a higher prevalence of mild infections in primary care doctor driven emergency systems and a higher prevalence of injuries in EDs have been reported [22]. This injury-focused activity in EDs has also been described elsewhere in all age groups [23, 24]. Thus, the present triplet of interventions seemed to shift the functions of the studied primary care ED towards the form of a standard specialized health care driven ED. Whether these low-acuity primary care services should then also be provided to the population out of office-hours is another question [25].

This was a retrospective study considering primary care EDs. As this study was purely register-based the subjects were not aware of their participation in the study. The present result reflects real clinical activity in this respect. As a confounding factor, electronic reminders were introduced in the electronic patient information system in 2008 to enhance recording of diagnoses and that may have altered the observed proportions of different diagnoses during the present study [26]. For example, this intervention explains at least partially the observed increase in symptomatic diagnoses (IDC-10 group R) during this study [26].

As a limitation, we should have been able to compare our results to a control city with a similar office-hours primary health care, demography, and size. This would have strengthened our conclusions. However, such data from another city were partially available for comparison only for the first two interventions [2, 3]. Data about possible changes in patient material or changes in ways to manage practices and diseases were not available. These factors have a considerable effect on changes in the number of visits to GPs. Data concerning these putative changes could have been obtained if we had had access to the patient information of individual patients. This would have allowed us to follow individual patients and their fates instead of plain numbers of visits. Furthermore, we would have needed data about how visits to the primary care nurses developed during the follow-up as the present type of interventions may shift patients from physicians to nurses [27]. Unfortunately, we were unable to obtain access to these data.

To our best knowledge, the health policy or system in Vantaa was not changed during the follow up but, naturally, we were not able to control all putative secular trends that can account for the observed reductions. For example, the population of Vantaa aged rapidly during the follow-up period [19]. This was the most obvious of these social trends but ageing of the population should not decrease the use of EDs. Cost savings were not observed as the health care costs increased linearly throughout the follow-up period [28].

## Conclusions

Decreasing services in a primary health care ED with the described interventions seemed to reduce the use of services by young people (0-19y). Especially visits due to respiratory diseases and mild infections were reduced but visits due to injuries increased. The present triplet of interventions seemed to make the primary care ED in this study look more like a standard ED driven by specialized health care.

## Data Availability

The data for the study is obtained from electronic patient chart system of Vantaa (Graphic Finstar); the authors do not have permission to share the original data with personification but anonymized raw data is attached with this publication.

## Abbreviations

ANOVA: Analysis of variance
ED: Emergency Department
SEM: Standard error of mean
ICD-10: International Classification of Diseases 10th edition
